# Text4Hope: An e-Mental Health Tool for Mitigating Psychological Symptoms among Young Adults

**DOI:** 10.1192/j.eurpsy.2024.174

**Published:** 2024-08-27

**Authors:** A. Belinda, R. Shalaby, W. Vuong, S. Surood, A. Greenshaw, A. Gusnowski, Y. Wei, V. I. O. Agyapong

**Affiliations:** ^1^Department of psychiatry, University of Alberta; ^2^Addiction and Mental Health, Alberta Health Services, Edmonton; ^3^Department of psychiatry, Dalhousie University, Halifax, Canada

## Abstract

**Introduction:**

Chronic stress, anxiety, and depression can interfere with young adults’ everyday function, academic achievement, and interpersonal relationships. Interventions aimed at preventing the deterioration or possibly onset of these mental disorders among young people are timely.

**Objectives:**

To assess the impact of a supportive text messaging program (Text4Hope) on the psychological well-being of young adults.

**Methods:**

This study adopted both longitudinal and naturalistic controlled trial designs. Longitudinal study: compared baseline and 6^th^ week outcomes in the same group of young adult subscribers. Naturalistic controlled study: compared clinical parameters in two groups of Text4Hope young adult subscribers: (i) intervention group (IG), subscribers who received once-daily supportive text messages for 6-weeks and completed 6th-week evaluation between 26 April and 12 July 2020, and (ii) control group (CG), subscribers who joined Text4Hope the same time frame, completed a baseline survey and were yet to receive text messages. The prevalence and severity of moderate-high stress, anxiety, and depression was measured using standardized scales. Inferential statistics, including the t-test, McNemar test, chi-square, and binary logistic regression analyses, were used to evaluate the differences in the prevalence and severity of the psychological symptoms.

**Results:**

Longitudinal study: subscribers who completed both the baseline and 6th-week surveys, had significant reduction in the prevalence of moderate-high stress (8%) and likely GAD (20%) from baseline to six weeks. The largest reduction in mean scores was for the GAD-7 scale (18.4%). Naturalistic study: significantly lower prevalence for likely Moderate Depressive Disorder (25.2%) and suicidal thoughts/thoughts of self-harm (48.4%), with a small effect size in the IG compared to CG.

**Image:**

**Figure:**
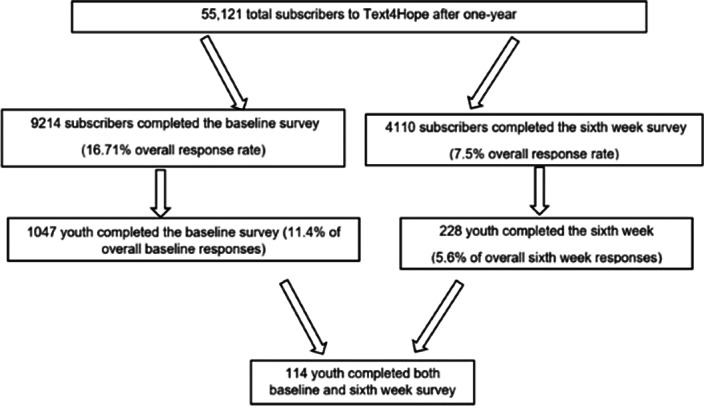

**Conclusions:**

TheText4Hope program has been demonstrated as an effective e-mental health tool for mental health support for young adult subscribers. This is particularly encouraging, as young adults have already adapted to SMS text messaging and texting. Therefore, this mode of intervention can be used to supplement existing treatments for psychological problems impacting young adults. In addition, the cost effectiveness and easy scalability of supportive text message interventions mean that policymakers and governments can quickly implement similar programs as part of national youth suicide prevention strategies.

**Disclosure of Interest:**

None Declared

